# Barriers to quality healthcare among transgender and gender nonconforming adults

**DOI:** 10.1111/1475-6773.14362

**Published:** 2024-07-10

**Authors:** Kedryn Berrian, Marci D. Exsted, Nik M. Lampe, Sayer L. Pease, Ellesse‐Roselee L. Akré

**Affiliations:** ^1^ The Dartmouth Institute for Health Policy and Clinical Practice, Geisel School of Medicine Lebanon New Hampshire USA; ^2^ Center for Applied Research and Educational Improvement, College of Education and Human Development University of Minnesota St Paul Minnesota USA; ^3^ Department of Mental Health Law & Policy University of South Florida Tampa Florida USA; ^4^ Department of Health Policy and Management Bloomberg School of Public Health Johns Hopkins University Baltimore Maryland USA

**Keywords:** gender nonconforming, gender‐affirming care, healthcare, nonbinary, transgender

## Abstract

**Objective:**

To determine the barriers transgender and gender nonconforming (TGNC) adults face when accessing or receiving healthcare in the United States.

**Data Sources and Study Setting:**

Primary data were collected between September 2022 and March 2023 from a purposive sample of TGNC adults (N = 116 participants) using an online survey with a series of open‐ended and closed‐ended questions.

**Study Design:**

Thematic analysis was employed to extract and analyze participants' responses to an open‐ended question about challenges they experienced when accessing or receiving healthcare. Two members of the research team conducted qualitative data analyses using Dedoose. The quality of each analysis was subsequently reviewed by a third research team member.

**Data Collection/Extraction Methods:**

Data were collected from responses to one open‐ended question that asked about participants' healthcare barriers as a TGNC individual.

**Principal Findings:**

Five main themes surrounding healthcare barriers emerged from the content analysis: (1) acceptability, (2) accommodation, (3) affordability, (4) availability, and (5) accessibility. First, participants who noted acceptability issues reported explicit discriminatory treatment from providers, providers not using their chosen names and pronouns (e.g., misgendering), and provider refusal to provide gender‐affirming care. Second, participants who experienced accommodation challenges identified provider medical training gaps on TGNC patient needs and administrative barriers to care. Third, participants explained affordability issues due to a lack of adequate health insurance coverage. Fourth, participants described availability challenges with accessing hormone therapy prescriptions. Finally, participants noted accessibility issues with obtaining TGNC‐specific care at LGBTQ+‐affirming clinics.

**Conclusions:**

There is a growing interest in the needs of TGNC adults within healthcare settings. This requires that health policies are enacted to ensure that TGNC adults have access to healthcare that is accommodating and accepting/affirming. Study findings may provide insight into the potential impact of current legislation on transgender access and availability.

**What is known on this topic**

- There is limited knowledge and training on the healthcare needs of transgender and gender nonconforming patients. This creates inequitable access to acceptable care and can contribute to health disparities within the population.

**What this study adds**

- This study adds evidence of the barriers and facilitators experienced by TGNC adults when accessing gender‐affirming care.
- Many TGNC respondents experienced discrimination and stigmatization when accessing healthcare.
- This study expounds on the existing literature by demonstrating that there are structural barriers to accessing care for TGNC adults.

## 1 INTRODUCTION

An estimated 1.3 million adults identify as transgender in the United States, and approximately 26% of this population identify as gender nonconforming.^1^ *Transgender* is an umbrella term referring to people who do not conform to their sex/gender assignment at birth (e.g., female/woman), and *gender nonconforming* is an umbrella term referring to people who do not conform to a social classification of the gender binary categories of women or men (e.g., nonbinary).^1^, ^2^ Deemed a National Institutes of Health disparity population, transgender and gender nonconforming (TGNC) communities face significant healthcare disparities in the United States.^3^, ^4^ Prior research notes a lack of access to quality healthcare for TGNC individuals due to key barriers, such as erasure in care settings, no or limited TGNC‐specific health services, and mistreatment by clinicians.^4^, ^5^ Indeed, healthcare professionals often lack sufficient medical knowledge and training in offering culturally responsive care to TGNC patients.^6^ Other care challenges include providers not using TGNC patients' chosen names and pronouns, gendered décor in patient exam rooms, TGNC stigma, and poorly designed electronic health records.^7^ These challenges create barriers to gender‐inclusive and safe TGNC care, increasing risks for adverse health outcomes and exacerbating healthcare inequities among TGNC people compared to their cisgender counterparts (i.e., those whose gender identity conforms with their sex/gender assigned at birth).^1^, ^8^

There is a critical need to fully address TGNC people's healthcare needs as a medically vulnerable population.^3^ TGNC Americans often experience various forms of structural erasure, stigma, and discrimination, especially when seeking and utilizing healthcare.^7^, ^8^ Many TGNC individuals, for example, report higher levels of gender discrimination within care settings than their cisgender peers.^4^, ^6^, ^7^, ^8^, ^9^ Healthcare professionals' transphobia was a stronger predictor for disparate healthcare experiences and outcomes than their lack of medical education regarding transgender patients.^10^ Previous research demonstrates how fostering inclusive and safe care environments for TGNC communities is an effective strategy to determine TGNC patients' precise needs and inform opportunities for targeted improvement in medical care.^11^ However, many US healthcare facilities do not have the infrastructure administratively to practice gender‐affirming care for TGNC patients (e.g., electronic health records that do not list TGNC patients' chosen names and pronouns), reflecting the structural barriers to providing quality TGNC care.^12^

Using Penchansky and Thomas' (1981) access to care conceptual framework, we examine the challenges that TGNC individuals face when accessing healthcare in the United States using the framework's five dimensions of accessibility: (1) availability, (2) acceptability, (3) accommodation, (4) affordability, and (5) accessibility.^13^ The five dimensions are defined in the following ways as they pertain to healthcare services: *availability*, the supply and distribution; *accessibility*, the geographic location and transportation; *accommodation*, the organization and operation; *affordability*, the cost and financing; and *acceptability*, the quality and appropriateness.^13^ Although prior studies have examined TGNC barriers to care, few have intricately examined their medical encounters and how these encounters subsequently influence their healthcare utilization.^4^, ^5^, ^6^, ^15^ While health insurance access has expanded, there are no national policies aimed at improving the cultural competency of healthcare professionals to ensure that TGNC patients receive appropriate and respectful healthcare. The purpose of this study was to better understand the experience of TGNC patients when accessing healthcare services. We hypothesize that there will be several barriers impeding access to healthcare services described by study participants. This study will add to the literature by providing insight into some of the experiences of TGNC patients when utilizing healthcare.

## 2 METHODS

### 2.1 Study design and participants

We collected data from a Qualtrics‐based online survey that employed a series of closed‐ended and open‐ended questions from a research sample of TGNC adults who reside in the United States. Data were extracted and analyzed from one open‐ended question: “Tell us about the challenges you've experienced when accessing or receiving healthcare.” We then used a thematic analysis approach to investigate the unique barriers to care faced by TGNC respondents. Survey respondents were recruited through snowball sampling via social media and purposive social network sampling strategies,^14^ wherein TGNC community leaders and organizations advertised the study to community members who may be interested in research participation. Eligibility included participants who: (a) self‐identified as transgender, nonbinary, and/or gender nonconforming; (b) were 18 years of age or older; (c) lived in the United States; and (d) consented to participate in the study. We also aimed to recruit a diverse set of TGNC respondents by race and ethnicity, socioeconomic status, sexual orientation, and geographical location. Each participant received a $5 incentive to complete the survey screener and an additional $15 gift card for completing the survey. This study was approved by the [university IRB].

### 2.2 Data collection

Data were collected using an online Qualtrics‐based survey with a series of closed‐ended and open‐ended questions, which addressed the following study aim: to understand barriers and facilitators to accessing health services and to understand the experiences of TGNC persons. Close‐ended questions collected data on research participant demographics and the types of health services, including gender‐affirming care services, that participants received. Additionally, 14 open‐ended questions in the online survey collected sociodemographic information on participants' as well as their experiences with identity document laws and policies to affirm their identities (i.e., changing gender markers on driver's license or passport). Examining one of the open‐ended question on TGNC healthcare barriers allowed us to assess participants' TGNC‐specific and unique challenges with accessing care from their own perspectives. This study offers a deeper understanding of the multilayered, healthcare barriers faced by TGNC adults in the United States.

Survey content was developed and informed by TGNC and other LGBTQ+ investigators from the study team to ensure questions were insightful, affirming, and culturally appropriate. The survey was distributed from September 2022 to March 2023. Once prospective participants were deemed eligible through a brief screening survey, they were required to consent to research participation via Qualtrics preceding the survey questions. This two‐part method helped to prevent online bots from completing the survey to access the gift card incentives.

### 2.3 Data review

There were 2283 total responses for the study, of those 2167 were spam or bot responses, leaving 116 verified survey responses from TGNC adults in the United States. To ensure the accuracy of the data sample, measures were taken to “clean” the data to eliminate bot participants (e.g., adding CAPTCHA questions). After the completion of the bot removal strategies, we removed all identifying information from participants' survey responses during the quality checking process to ensure research participant confidentiality. The entire survey presented participants with substantive questions concerning their (i) healthcare experiences, (ii) barriers to gender‐affirming care, and (iii) health needs and management. For the purpose of this study, we focused on one question focusing on participant healthcare experiences.

### 2.4 Data analysis

Two investigators from the study team coded open‐ended responses using Dedoose (9.0.107) software and analyzed the data inductively, utilizing a thematic analysis approach. The study team developed a codebook. The coder team first developed a detailed codebook, which consisted of a series of codes grounded in evidence‐based knowledge on TGNC barriers to care (e.g., accommodation). Additional codes were then identified during the initial coding stage of data analysis. Responses to the following question were thematically coded: “Tell us about the challenges you've experienced when accessing or receiving healthcare.” Qualitative thematic analysis was utilized to interrogate the responses from participants.

All survey responses to the research question were reviewed independently and analyzed by two investigators, KB and ME. KB and ME engaged in initial or open coding, meaning they read a subset of survey responses to develop a general sense of the data and generated an initial list of codes based on Penchansky and Thomas's (1981) access to care framework.^13^ Subsequently, they engaged in focused or thematic coding, resulting in the combining and collapsing of open codes to form broader thematic codes/themes. An additional team member conducted a quality check on the analyses, and all study members concurred on the data analysis and findings from the open‐ended survey responses to the question: “Tell us about the challenges you've experienced when accessing or receiving healthcare.”

## 3 RESULTS

### 3.1 Study sample

The final study sample consisted of 116 TGNC adults who reside in the United States. Respondents ranged from 18 to 55 years of age (median age: 29.5 years). Regarding race/ethnicity, 17 participants (14.7%) self‐reported as multiracial, 3 (2.6%) as Black and/or African American, 3 (2.6%) as Hispanic/Latino, 2 (1.7%) as Asian, 1 (0.9%) as Indigenous, 1 (0.9%) as Middle Eastern/North African, and 89 (76.7%) as White, non‐Hispanic/Latino. We incorporate race and ethnicity as variables in this study due to the well‐documented instances of structural racism among racial and ethnic minority TGNC communities, especially Black TGNC people, when accessing and utilizing healthcare. Sixty percent of respondents identified as transmasculine (i.e., an individual who was assigned female at birth but identified and lived as masculine indiscriminate of medical transitioning). Most of the respondents (43.97%) identified as having multiple sexual orientation identities. More than 28% have a 4‐year college degree and more than 37% of respondents reported their annual individual earnings less than $25,000 annually. Nearly 60% of respondents had full‐time employment and 68% of respondents had employer–sponsored insurance. Lastly, nearly 39% of respondents were from the Western US region.

### 3.2 Thematic themes

Five themes were derived from the thematic analysis of the qualitative data. Acceptability and accommodation were the most common access concepts found, including the ways these themes intersected across experiences shared by TGNC participants. The acceptability theme was divided into three subthemes: *systematic dehumanization in care settings*, *deadnaming and misgendering*, and *refusal to provide equitable care*. The accommodation theme was divided into two subthemes: *lack of TGNC healthcare training and awareness* and *administrative barriers to care*. The affordability theme was assessed under one subtheme: *lack of health insurance coverage*. The availability theme was assessed under one subtheme: *insufficient access to hormone therapy prescriptions*. Finally, the accessibility theme was accessed under one subtheme: *inaccessibility in obtaining TGNC‐specific care at LGBTQ‐affirming clinics*. Table 1 lists the main themes and subthemes extracted from the qualitative data.

**TABLE 1:** Main themes and subthemes extracted from qualitative data.

Themes	Subthemes
1. Acceptability	1.1 Systematic dehumanization in care settings 1.2 Deadnaming and misgendering 1.3 Refusal to provide equitable care
2. Accommodation	2.1 Lack of TGNC healthcare training and awareness 2.2 Administrative barriers to care
3. Affordability	3.1 Lack of health insurance coverage
4. Availability	4.1 Insufficient access to hormone therapy prescriptions
5. Accessibility	5.1 Inaccessibility in obtaining TGNC‐specific care at LGBTQ+‐affirming clinics

### 3.3 THEME 1: Acceptability

#### 3.3.1 Subtheme: Systemic dehumanization in care settings

In a healthcare setting, I never quite know how I'm going to be treated.

The primary challenge faced by TGNC adults was explicit discriminatory treatment by providers and clinic staff, emboldened by systemic structures of oppression. Responses disclosed how medical professionals diminished participants' TGNC identities. A participant explained,

It's hard to go to the ER or urgent care because I frequently get misgendered and asked questions that are quite inappropriate, such as “Do you still have your parts?” [Participant 42]

Consequently, many participants reported feelings of uncertainty about the quality of their medical care due to medical providers and staff invalidating respondents' experiences during clinical encounters. A respondent described their experience in obtaining an attention deficit/hyper activity disorder diagnosis evaluation:

The doctor completely blew me off, ignored everything he didn't want to see, put nonbinary in quotes in his report like it wasn't a real thing. In a healthcare setting, I never quite know how I'm going to be treated. [Participant 17]

Such experiences with systemic dehumanization in care settings serve as a deterrent for TGNC patients by discouraging them from accessing and maintaining health services.

#### 3.3.2 Subtheme: Deadnaming and misgendering

It is vulnerable enough to go to a doctor and then to be misgendered on top of that.

About 25% of respondents reported being deadnamed or misgendered, often intentionally, by medical providers and staff. *Misgendering* involves using pronouns or gendered language that does not align with a person's gender identity, while *deadnaming* refers to using a name that a TGNC person no longer uses, such as their name assigned at birth. Such instances still occurred even after respondents offered multiple requests for healthcare professionals to respect their chosen pronouns and names. A participant faced this treatment during a vulnerable time in their life:

When I was hospitalized for a suicide attempt, the doctors and nurses in that particular institution refused to call me by my preferred name and made sure to treat me as female. [Participant 5]

Participants also conveyed how the absence of knowledge or awareness regarding TGNC experiences among medical providers conveys to them the impression that their patient needs will not be adequately addressed during clinical encounters:

They also don't believe that trans people exist so when I go they do not use my pronouns or know enough about my medical history to be much help. [Participant 40]

#### 3.3.3 Subtheme: Refusal to provide equitable care

Providers have declined to provide me care.

In addition to discriminatory treatment during care, some of our participants experienced being denied care outright due to their gender identity. Respondents noted:

When I first came out my PCP refused to care for me, and I had to switch. [Participant 47]

Several providers have questioned the validity of my gender identity. Some providers have declined to provide me care due in part or in whole to my gender identity. [Participant 56]

For some, denial of care experiences occurred outside of clinical settings as well, with one participant having their prescription unduly denied:

The most challenging experience I've had was trying to get my HRT [Hormone Replacement Therapy] from the pharmacy. They misgendered me constantly and would not fill the prescription according to how my doctor prescribed it. I finally had to transfer pharmacies and haven't had a problem since. [Participant 73]

### 3.4 THEME 2: Accommodation

#### 3.4.1 Subtheme: Lack of TGNC healthcare training and awareness

Respondents described barriers with providers' limited training on TGNC healthcare needs and awareness of TGNC patients. This lack of knowledge often required TGNC participants to take an educator role during medical encounters. This training gap included providers' assumptions about patients' TGNC experiences presenting in a medicalized manner. Clinicians frequently presumed that being TGNC required taking hormones, to medically transition, or that it exclusively referred to identifying as a transgender woman or man:

Just annoying educating primary care providers about my name/pronouns/nonbinary things in general when they asked if I had any surgeries and explaining why I had top surgery and that no, I'm not interested in HRT or a transition that looks like a binary trans man. [Participant 101]

I tried to talk about my frustration with my dysphoria with my therapist and she did not understand. I never got to address anything I wanted to address and wound up educating her on gender fluidity. [Participant 100]

One participant recounted an experience where a therapist was unhelpful, as they could not provide any documentation for the participant's transition:

I had a therapist that told me she was able to write a letter of support, so I saw her for a year and a half and when I asked about it, she told me that she could not give me one (or anyone else because of her lack of experience with trans clients). This obviously delayed my transition. [Participant 83]

#### 3.4.2 Subtheme: Administrative barriers to care

Participants detailed encountering administrative hurdles that hindered their access to and maintenance of healthcare, affecting their well‐being:

I am not able to add my pronouns to my own medical record. I had to add them to my Preferred Name category and that makes me feel less valued. [Participant 39]

Even in states that allow for nonbinary identification, respondents reported how care facilities only allowed for two sex/gender categories in patients' electronic health records, creating logistical challenges for them receiving TGNC care:

I have had issues with my insurance stating I'm nonbinary but the medical places only having male or female and they make me choose in order to put it in their system. When it doesn't match[,] the[n] issues arise with insurance coverage. [Participant 73]

One person explained how larger systemic issues of the healthcare workforce bleed into clinical environments:

Not feeling like the doctor has the time to really listen and attend—like the doctor's office is a factory setting. [Participant 15]

The comparison of the doctor's office to a “factory setting” implies a depersonalized and transactional healthcare environment, where patients might feel like mere numbers in a system rather than individuals with unique needs and concerns. Other participants expressed similar concerns about receiving patient‐centered care as TGNC adults.

### 3.5 THEME 3: Affordability

I am in debt because of my gender identity not being something that people deem real.

#### 3.5.1 Subtheme: lack of health insurance coverage

Many participants expressed that many of their healthcare needs (whether related to gender‐affirming care or not) were financially inaccessible due to insufficient or nonexistent health insurance coverage. Some reported that their health insurance policies did not consider their TGNC healthcare needs, particularly gender‐affirming surgeries, eligible for coverage:

My insurance keeps denying to cover my body masculinization [surgery] and saying it's not medically necessary in my ‘case’ to treat gender dysphoria which is completely false and discriminatory. [Participant 113]

Another participant explained that their health insurance inadequately covers gender‐affirming surgeries. They faced not only financial constraints but also lacked the energy, resources, and knowledge necessary to advocate for comprehensive insurance coverage:

I live in Arizona, and the Medicaid (charter?) here explicitly does not cover gender affirming surgical interventions. I am told, however, that some people have been able to negotiate it, but I don't have any fight in me to get through this anymore. The system is confusing, and it seldom offers help. [Participant 18]

### 3.6 THEME 4: Availability

#### 3.6.1 Subtheme: insufficient access to hormone therapy prescriptions

TGNC participants expressed their difficulties receiving prescriptions, noting instances of supply shortage or denial by healthcare providers. A common issue participants reported was the denial of hormone therapy prescriptions from healthcare providers. In recent years, pharmaceutical companies across the United States have faced challenges in reliably ensuring the availability of injectable estrogen.^16^ This critical issue has been considered a public health crisis for TGNC patients since hormone therapies are medically necessary interventions for many individuals within this community.^16^ One participant highlighted frequent shortages of their estrogen medication:

Estradiol valerate, which I inject weekly, is constantly threatened by supply shortage. [Participant 18]

Participants reported how denial of hormone therapy prescriptions from healthcare providers arises from various factors, including limited medical training and awareness about TGNC healthcare needs, resulting in inadequate or unreliable prescription practices for TGNC patients who seek gender‐affirming care. A respondent noted that the denial of the prescriptions delays their hormone therapy services:

Often prescriptions are denied and I have to wait even longer to get HRT. [Participant 105]

### 3.7 THEME 5: accessibility

#### 3.7.1 Subtheme: inaccessibility in obtaining TGNC‐specific care at LGBTQ+‐affirming clinics

Participants expressed challenges in obtaining TGNC‐specific care via gender‐affirming medical interventions at clinics that were widely known or advertised as LGBTQ+‐affirming. One TGNC participant discussed their experience with the unpredictability of accessing gender‐affirming care after receiving a referral from a community member:

One time when I was looking for a new provider after moving states with my wife, I went to see a provider and after the appointment they told me they were no longer doing gender affirming care. [Participant 66]

Other TGNC patients described the policy barriers to receiving TGNC‐specific care in LGBTQ+‐friendly clinical environments due to their states' gender‐affirming care bans or severe restrictions:

Getting access to HRT was the most challenging thing of my transition so far. The nearest Planned Parenthood to me is an hour drive and they do not provide HRT to people in my state, most online services like folx are unable to prescribe HRT online due to my state[’]s regulations. [Participant 114]

## 4 DISCUSSION

The purpose of this study was to examine the unique challenges TGNC adults in the US face when accessing and utilizing care. Study findings demonstrated that TGNC people experience several barriers to healthcare access across all five dimensions of access. Several respondents reported that they were not able to obtain acceptable care, as the care that they received did not meet their expectations of quality or appropriateness. This is consistent with previous studies that provide evidence of the mistreatment and experiences of discrimination in TGNC people when seeking healthcare.^7^, ^8^ The lack of accommodation and acceptability within healthcare settings is supported by the present study. Participants cited their healthcare providers' ignorance and assumptions about their sexual behavior; they noted that healthcare providers' lack of knowledge and transphobic comments made them feel uncomfortable, especially when those questions were intrusive and irrelevant to care. These comments are explicitly inappropriate and expand our understanding of how providers, regardless of their intentions, can make TGNC patients feel unwelcomed or unsafe in healthcare settings. Healthcare providers should ensure gender inclusivity and affirmation for their TGNC patients during clinical encounters by avoiding inappropriate questions stemming from misinformation and implicit biases.

When discussing experiences of discrimination and stigmatization, it is important to acknowledge intersecting systems of cumulative disadvantage (e.g., TGNC identity and low socioeconomic status) also substantially exacerbate healthcare barriers among TGNC communities.^17^ Because TGNC people of color experience a unique type of discrimination due to their social identities simultaneously targeted by racism and transphobia, many TGNC patients of color have reported being mistreated by medical providers and staff, while struggling to access culturally tailored healthcare.^15^, ^17^ Many transgender patients of color have reported substantial concerns with medical racism when seeking LGBTQ+‐friendly providers and prior negative healthcare interactions with medical providers that were attributed to their racial and or ethnic minority identity.^18^ Thus, assessing intersectionality among TGNC patients—in relation to TGNC barriers of care—is crucial when addressing TGNC people's healthcare disparities.^17^

Affordability was another significant barrier to care. Participants frequently reported financial reasons for forgoing or delaying care. In the study by Carpenter et al., TGNC adults are more likely to be in poverty than their cisgender peers, which was also illustrated in our data.^19^ Access to adequate health insurance can provide many TGNC adults the necessary care to receive gender‐affirming care and other healthcare needs.^19^ Our results revealed that the main barriers to healthcare access are long wait times, particularly for medications, and a lack of inclusive providers available for appointments. Finally, it is important to acknowledge that for many TGNC patients, financial insecurity and the absence of insurance coverage persist as structural barriers to access high‐quality care in the United States, a country with no universal healthcare system.^20^ For instance, many gender‐affirming medical interventions (e.g., gender‐affirming surgeries and hormone therapies) are not covered by health insurance.^21^ Lack of access to gender‐affirming medical interventions for TGNC people can prompt additional negative health outcomes (e.g., suicidal ideation) and may increase instances of medical harm.^22^ This has been somewhat mitigated by The Affordable Care Act, a US‐based federal legislation that provides affordable health insurance to socioeconomically disadvantaged persons, including gender‐affirming medical interventions.^23^ However, TGNC communities still face additional financial challenges when accessing quality care.

Healthcare settings should utilize inclusive practices and language. For TGNC adults, recognizing appropriate terms for gender identities is important to create a safe healthcare experience.^24^ In electronic medical records, it is recommended that healthcare providers use accurate language that describes TGNC patients. Terms such as “female‐to‐male” (FTM) and “male‐to‐female” (MTF) can be considered a bit antiquated and emphasize a medicalized process. Terms such as “transfeminine” and “transmasculine” are more popular and emphasize the direction of transness.^12^

### 4.1 Study strengths and limitations

There were notable strengths and limitations in this study. First, TGNC participants primarily provided thematically rich responses to the open‐ended survey question: “Tell us about the challenges you've experienced when accessing or receiving healthcare.” This provided a comprehensive understanding of the intricate healthcare experiences of TGNC adults as well as their unique challenges in accessing and maintaining adequate care. Additionally, we conducted a rigorous data review and analysis process, using thematic analysis approaches to uncover the themes noted in this research study. Regarding study limitations, our findings are not generalizable to the general TGNC population, especially concerning the racial/ethnic generalizability of the research sample. Such limitation may be due to snowball sampling strategies in survey data collection, which limited the racial/ethnic diversity of the research sample. Finally, the study did not specify what type of healthcare, which prevents this study from being attributed to certain specialties within the US healthcare system.

## 5 IMPLICATIONS FOR HEALTH SERVICES RESEARCH AND POLICY

Findings from the study demonstrate that there are several barriers to accessing care for TGNC patients. These barriers are multilayered ranging from interpersonal (i.e., limited cultural competency of providers, implicit biases, and experiences of discrimination), limitations in internal infrastructure (i.e., health records management systems that facilitate misgendering and deadnaming), and policy and systematic (i.e., limitations of health insurance coverage). The shared experiences of participants underscore the need for comprehensive reforms at multiple levels to remove barriers to accessing high‐quality, safe, and appropriate care for TGNC individuals. Comprehensive reform in medical education and licensure could improve knowledge about TGNC patient needs and reduce implicit biases toward the population. This change could reduce instances of discrimination when accessing healthcare services within this population. Standardized collection of sexual orientation and gender identity data should be used within all healthcare practices to ensure that internal systems like electronic health records and electronic medical records are not contributing to the misgendering and dead naming of TGNC patients. The reasoning for the collection of this data should be clearly articulated to patients and should be secured to ensure the safety and privacy of the patients. Recommendations for how to collect SOGI data can be found in the literature.^25^, ^26^

Standards of minimum coverage should be established to ensure that TGNC patients' healthcare needs are included. Similarly, to how the Patient Protection and Affordable Care Act requires Marketplace health plans to provide free preventative care benefits for all adults, women, and children, certain gender‐affirming care services should, at the very least, be included in health insurance benefits with a reasonable co‐pay or co‐insurance costs.^27^ Providing some coverage for gender‐affirming care services, such as mental health services, hormone replacement therapy, surgical interventions, hair removal and management, voice and communication therapy, primary care, and preventative health screenings, would remove the financial barrier to lifesaving health services for many TGNC patients.

## 6 CONCLUSION

Given existing healthcare disparities among TGNC communities in the United States, there is an urgent need for comprehensive reforms in US healthcare education, policy, and practice to address the systemic issues that hinder access to culturally responsive and inclusive care for TGNC patients.^3^ Our findings highlight acceptability (e.g., deadnaming and misgendering practices), accommodation (e.g., provider training gaps on TGNC patient needs), affordability (e.g., lack of health insurance coverage for gender‐affirming medical interventions), availability (e.g., insufficient access to hormone therapy prescriptions), and accessibility (e.g., challenges with obtaining TGNC‐specific care at LGBTQ+‐affirming clinics) barriers to receiving quality care for TGNC patients, driving existing inequities within US healthcare systems. Research and community partners should continue to focus on increasing TGNC healthcare training and awareness among healthcare workers and environments, updating electronic health records to reflect TGNC patients' chosen pronouns and names, and affordable options for accessing gender‐affirming medical interventions. Eliminating such barriers is essential for achieving health equity in TGNC communities and ensuring that everyone has unimpeded access to gender‐affirming care.

## FUNDING INFORMATION

Funding was provided for this project from the Geisel Center for Health Equity at Dartmouth College and the Robert Wood Johnson Health Equity Scholars for Action career development award (#81412).
